# Uncommon Entities, Uncommon Challenges: A Review of Rare Plasma Cell Disorders

**DOI:** 10.3390/hematolrep17040031

**Published:** 2025-06-27

**Authors:** Amr Hanbali, Abdullah Alamer, Saud Alhayli

**Affiliations:** Department of Hematology and Cellular Therapy, King Faisal Specialist Hospital & Research Center, Riyadh 11211, Saudi Arabia; aalamer2@kfshrc.edu.sa (A.A.); salhayli@kfshrc.edu.sa (S.A.)

**Keywords:** rare plasma cell disorders, IgD multiple myeloma, IgE multiple myeloma, IgM multiple myeloma, non-secretory myeloma, plasma cell leukemia, heavy chain disease, diagnostic challenges

## Abstract

Rare plasma cell disorders—including IgD, IgE, and IgM multiple myeloma, non-secretory myeloma (NSMM), plasma cell leukemia (PCL), and heavy chain disease (HCD)—are biologically heterogeneous and often present with atypical features and aggressive behavior. This review synthesizes current evidence on their epidemiology, pathophysiology, diagnosis, and treatment. Advances in proteasome inhibitors, immunomodulatory agents, and autologous transplantation have improved outcomes in select subtypes. However, challenges persist in distinguishing IgM myeloma from Waldenström macroglobulinemia, monitoring non-secretory disease, and treating highly aggressive forms such as IgE myeloma and PCL. Standardized diagnostic criteria and prospective trials are essential to guide future management.

## 1. Introduction

Plasma cell disorders represent a heterogeneous group of clonal disorders originating from terminally differentiated B cells, characterized by the production of a monoclonal immunoglobulin (Ig) or its subunits [1]. These disorders range from indolent conditions such as monoclonal gammopathy of undetermined significance (MGUS) to aggressive malignancies including multiple myeloma (MM) and plasma cell leukemia (PCL) [2]. MM, the most prevalent form, is a malignancy of bone marrow plasma cells and is typically associated with end-organ damage. Its classic clinical features are summarized by the acronym SLiM-CRAB, which includes both traditional myeloma-defining events—hypercalcemia, renal failure, anemia, and bone lesions (CRAB)—as well as newer biomarkers: ≥60% clonal plasma cells in bone marrow (S), involved/uninvolved free light chain ratio ≥ 100 (Li), and >1 focal lesion on MRI (M). The diagnosis of MM requires ≥10% clonal plasma cells in the bone marrow in combination with at least one CRAB feature or a myeloma-defining event [3]. Although MM accounts for approximately 1% of all cancers, it is the second most common hematologic malignancy after lymphoma. The global five-year prevalence of MM is estimated at around 230,000 patients [4].

In current clinical practice, the management of common MM subtypes—namely, IgG, IgA, and light chain MM, which account for approximately 52%, 21%, and 16% of cases, respectively—is guided by well-established evidence-based protocols [5]. In contrast, evidence on rare MM subtypes, such as IgD, IgE, and IgM myeloma, as well as non-secretory myeloma, PCL, and heavy chain disease, is limited. Information on these entities is primarily derived from isolated case reports and small case series, leading to ongoing challenges in diagnosis and a lack of consensus on optimal management strategies within the hematology community. Importantly, the fifth edition of the World Health Organization (WHO-HAEM5) recognize these entities within the broader spectrum of plasma cell neoplasms, but with nuanced differences in categorization. For instance, PCL is now formally subclassified as primary (de novo) or secondary (evolving from MM), and NSMM is acknowledged as a diagnostically challenging form within MM variants due to lack of measurable monoclonal protein. While IgD, IgE, and IgM MM are considered immunoglobulin-isotype-defined variants of MM, HCD is categorized under mature B-cell neoplasms with plasmacytic differentiation, rather than classic MM [6]. These classifications underscore the biological heterogeneity of rare plasma cell disorders and highlight the need for refined diagnostic and therapeutic frameworks.

This article aims to provide a comprehensive review of the current literature concerning these rare plasma cell disorders, highlighting diagnostic nuances, clinical behavior, and emerging treatment paradigms.

## 2. IgD Multiple Myeloma

IgD MM (Table 1) is a rare and biologically distinct subtype of plasma cell disorder, accounting for only 1–2% of all MM cases worldwide. First described by Rowe and Fahey in 1965, it is characterized by aggressive clinical behavior, lambda light chain restriction, and a tendency to present at an advanced stage with extensive organ involvement including renal dysfunction and amyloidosis [7,8]. Due to the inherently low serum concentration of IgD and the subtle or absent M-protein spike on standard serum protein electrophoresis, diagnosis is often delayed or missed unless specific immunofixation or free light chain assays are employed [9]. Patients with IgD MM typically present at a younger age than those with more common MM subtypes, often between 50 and 60 years, and more frequently in males [8,10].

Clinical features commonly include renal impairment, Bence Jones proteinuria, bone lesions, hypercalcemia, and systemic amyloidosis [11,12]. A multicenter Asian study involving 356 patients emphasized the high frequency of renal insufficiency, hypercalcemia, and advanced International Staging System (ISS) stage at presentation in IgD MM compared to non-IgD subtypes [7]. Lambda light chains predominate in over 85% of cases [10,13]. As mentioned earlier, due to low serum IgD levels (normal range: 0–10 mg/dL), standard electrophoresis often fails to detect a monoclonal peak, necessitating advanced diagnostic tools such as immunofixation, serum free light chain assays, or cytoplasmic IgD flow cytometry for accurate diagnosis [9,14]. In a recent Italian retrospective study of 15 patients with IgD MM, monoclonal protein was undetectable on serum protein electrophoresis in 60% of cases, highlighting the limitations of traditional diagnostic techniques [7].

IgD MM often exhibits complex cytogenetics, including deletion 13q, IGH rearrangements; 1q21 amplification; and, less commonly, high-risk abnormalities such as t(4;14), t(14;16), and deletion 17p [7,10,15]. In the Asia Myeloma Network (AMN) study, 40.6% of IgD cases had cytogenetic abnormalities, with t(11;14) occurring in 29%—significantly more than in non-IgD subtypes [16]. Although deletion 17p is a high-risk feature in MM, its prognostic impact in IgD MM varies, with some cohorts showing no consistent association with inferior survival [15]. A Chinese cohort reported higher rates of 1q21 amplification and t(14;16) in IgD patients compared to others, both linked to poor outcomes [10]. Nonetheless, cytogenetic profiling in IgD MM remains limited and heterogeneous, highlighting the need for prospective studies to refine risk stratification.

Historically, IgD MM was considered to confer a poor prognosis, with median overall survival (OS) ranging between 13 and 21 months in early studies [8,13]. This was largely attributed to delayed diagnosis, high tumor burden, and a greater prevalence of renal failure and other adverse features at presentation. However, more recent data suggest that the prognosis of IgD MM has significantly improved due to the use of targeted therapies and autologous stem cell transplantation (ASCT). For instance, the UK National Trials Group reported a substantial increase in median OS from 22 months (1980–2002) to 48 months (2002–2016) among patients with IgD MM, coinciding with the adoption of thalidomide, lenalidomide, and bortezomib into frontline therapy regimens [10]. Similarly, the Greek Myeloma Study Group found no significant survival difference between IgD and non-IgD MM patients treated in the era of first generation novel agents, with both groups achieving median OS exceeding 50 months [11]. Nevertheless, renal insufficiency remains a strong negative prognostic factor. In a prospective cohort study from Zhengzhou University, 55% of newly diagnosed IgD MM patients presented with renal dysfunction, which was independently associated with a significantly shorter OS (29 months vs. >40 months in those without renal impairment) [15]. Beta-2 microglobulin was identified as a reliable predictor of renal damage in these patients, emphasizing the importance of early biochemical monitoring. These regional discrepancies in survival may reflect differences in healthcare infrastructure, access to novel therapies, supportive care strategies, and underlying genetic or biological heterogeneity among patient populations. Consequently, direct comparisons across cohorts should be interpreted cautiously, and they highlight the need for harmonized, prospective international studies.

The therapeutic landscape of IgD MM has evolved significantly with the introduction of proteasome inhibitors, immunomodulatory drugs, monoclonal antibodies, and stem cell transplantation. The AMN cohort demonstrated that patients receiving targeted therapies had superior OS compared to those treated with conventional chemotherapy [7]. The response rate following induction therapy in more recent UK trials reached as high as 89%, compared to only 43% in earlier studies [10]. Despite these improvements, real-world outcomes can vary. A Chinese single-center study reported inferior survival among IgD patients despite access to novel agents, with median OS remaining below 25 months, suggesting that late-stage presentation and high-risk biology may still override treatment benefits in some cases [16]. A notable gap in the current evidence is the absence of prospective trials specifically evaluating quadruplet-based induction regimens, now considered standard in newly diagnosed MM, in the IgD MM population. As a result, it remains uncertain whether treatment recommendations for conventional MM can be universally applied to this rare subtype. In clinical practice, most patients with IgD MM are nonetheless managed using regimens such as Dara-VTd or Dara-VRd, similar to approaches in other high-risk or rare variants like IgE MM. This highlights the urgent need for subtype-specific prospective studies to determine the efficacy and safety of intensified combination therapies, and to inform evidence-based guidelines tailored to IgD MM. Furthermore, early diagnosis, risk-adapted strategies, and inclusion of IgD MM in trials investigating next-generation immunotherapies, such as CAR T-cell therapy and bispecific antibodies, remain critical priorities.

In conclusion, IgD MM is a rare but clinically aggressive subtype of MM with unique biological and clinical features. While historically associated with dismal outcomes, recent data indicate that the incorporation of novel therapeutic agents and ASCT has markedly improved survival. However, the disease remains challenging due to diagnostic delays, high rates of renal impairment, and complex cytogenetic profiles. Advances in diagnostic precision, risk-adapted therapy, and prospective clinical trials are essential to further optimize outcomes, guide therapeutic decision making, and develop consensus-based management strategies tailored to this rare and understudied plasma cell disorder. Key unanswered questions include whether IgD MM harbors distinct molecular drivers that may be targetable beyond current MM therapies, and how novel immunotherapies, such as bispecific antibodies and CAR T-cell constructs, can be effectively integrated into treatment paradigms for patients with high-risk IgD MM phenotypes. Dedicated translational studies and inclusion of IgD MM in biomarker-driven trials are warranted to address these gaps.

## 3. IgE Multiple Myeloma

IgE MM (Table 2) is the rarest immunoglobulin subtype of MM, accounting for fewer than 0.1% of cases. Since its first description by Johansson and Bennich in 1967, few case reports and small series have been published, making comprehensive understanding and evidence-based management challenging. Compared to more common isotypes like IgG and IgA, IgE myeloma exhibits a more aggressive clinical behavior, with frequent extramedullary involvement, PCL, and rapid progression, often resulting in inferior outcomes and shorter survival times [9,17,18].

The clinical presentation of IgE MM mirrors other MM subtypes in terms of anemia, bone pain, hypercalcemia, renal impairment, and osteolytic lesions. However, some studies suggest a higher incidence of aggressive features such as PCL and hyperviscosity syndrome [19,20,21]. In one of the earliest series, IgE MM was frequently associated with male sex and younger age at onset, and patients often presented with a severe disease burden [9]. A rare presentation involving both IgE and IgA monoclonal proteins in the same patient has also been reported, with molecular analysis confirming a common clonal origin of the dual-secreting plasma cells [22].

Laboratory diagnosis remains complex, as the low concentration of IgE in serum often leads to false-negative results in standard protein electrophoresis. Immunofixation using IgE-specific antisera and serum free light chain assays are essential for accurate detection. In certain cases, using undiluted serum samples or repeat testing may be required to confirm the diagnosis [23,24]. A case described by Altinier et al. highlighted the inconsistency between immunofixation and immunometric techniques, suggesting that IgE degradation or modification during renal filtration could interfere with epitope recognition [25].

IgE MM may infrequently arise from MGUS or progress to secondary PCL. Galakhoff et al. reported a case with IgE MGUS that eventually transformed into IgE-producing PCL over several years, underscoring the need for long-term vigilance in such patients [19]. PCL is the most aggressive manifestation among plasma cell disorders and carries a median survival of only one to two months in its secondary form [19].

Another uncommon but clinically important complication of IgE MM is hyperviscosity syndrome, which may arise due to markedly elevated IgE levels in the serum. Proctor et al. documented a case where IgE paraproteinemia led to classical signs of hyperviscosity syndrome and required urgent medical intervention [20]. Moreover, elevated CA125—a tumor marker commonly associated with epithelial ovarian cancer—has been reported in IgE myeloma without underlying solid malignancy, indicating potential cross-reactivity or paraneoplastic phenomena [18].

Recent studies have provided insights on the biology of IgE plasma cells. Using murine models and single-cell RNA sequencing, Vecchione et al. showed that IgE plasma cells are transcriptionally distinct from other isotypes. Short-lived IgE plasma cells arise early during antigen exposure in lymphoid tissues, while long-lived cells accumulate in the bone marrow after prolonged stimulation. These cells express high levels of protein synthesis, endoplasmic reticulum stress, and survival markers like TACI and BCMA, supporting their persistence and high immunoglobulin secretion [24]. A case report by Kehl et al. revealed a hypermutated phenotype in IgE MM, suggesting potential for neoantigen-directed immunotherapy [25]. These discoveries position IgE MM as both a clinical rarity and a model for immunogenetic research and targeted treatments.

There is no standardized treatment protocol specifically designed for IgE MM due to its rarity. However, most patients are managed using regimens used for conventional MM, including proteasome inhibitors, immunomodulatory drugs, and autologous stem cell transplantation (ASCT). Some cases have shown durable remissions with modern triplet or quadruplet induction followed by ASCT, while others progressed rapidly despite aggressive therapy [18,26]. Overall, reported median survival is shorter than that observed in IgG or IgA myeloma, though this gap may be narrowing with the availability of novel agents [9,24].

IgE multiple myeloma is an exceptionally rare and biologically distinct variant of plasma cell myeloma. It poses significant diagnostic challenges due to its subtle laboratory profile and low serum immunoglobulin concentration. Clinically, it often presents aggressively, with features like PCL or hyperviscosity syndrome. However, ongoing advances in molecular diagnostics, immunogenomics, and therapeutic strategies hold promise for improving the management and prognosis of affected patients. Increased awareness and case documentation are needed to enhance our understanding of this enigmatic disease. Important areas for future investigation include defining the distinct immunogenetic signatures or microenvironmental factors that may drive IgE MM pathogenesis and therapeutic resistance. Evaluating the potential utility of targeted treatments, particularly those directed against plasma cell antigens such as BCMA, represents a promising yet unexplored avenue.

## 4. IgM Multiple Myeloma

IgM MM (Table 3) is an exceptionally rare plasma cell disorder, constituting less than 0.5% of all MM cases. Its clinical presentation often overlaps with Waldenström macroglobulinemia (WM), a more common B-cell lymphoproliferative disorder that also secretes IgM. Due to overlapping features, accurate differentiation is critical for therapeutic decisions and prognostication [26,27].

IgM MM is characterized by a clonal proliferation of plasma cells producing IgM paraprotein, with ≥10% plasma cell infiltration in the bone marrow and typical MM-related organ damage (CRAB features: hypercalcemia, renal dysfunction, anemia, bone lesions). The presence of lytic bone lesions or translocation t(11;14) further supports the diagnosis [28,29]. Cytogenetically, IgM MM frequently harbors t(11;14), leading to cyclin D1 dysregulation. This alteration, detected in up to 39–50% of cases, is absent in WM and supports the diagnosis of IgM MM over WM [28,30]. Immunophenotypically, IgM MM plasma cells may show expression of CD38 and cyclin D1, but lack CD20, CD56, and CD117, distinguishing them from WM clones [31].

Patients typically present with MM features—bone pain, anemia, renal impairment, and hypercalcemia. A monoclonal IgM protein is typically detected. However, WM-like manifestations (lymphadenopathy, hepatosplenomegaly, hyperviscosity) can coexist, complicating the diagnosis [26,32]. Histologically, WM shows lymphoplasmacytic infiltration, whereas IgM MM reveals sheets of plasma cells [29]. Immunophenotyping and mutational profiling aid in differentiation: IgM MM lacks the MYD88 L265P mutation (>90% in WM), aiding differentiation, along with t(11;14), lytic lesions, and immunophenotypic markers (CD20^−^CD56^−^CD117^−^) [29,30,31,32,33]. A case report described spinal cord compression from plasmacytomas in IgM MM [34].

Castillo et al. conducted the largest retrospective analysis of 134 patients with IgM MM and found a median overall survival (OS) of 61 months, with higher ISS stage correlating with poorer outcomes [28]. This survival is comparable to non-IgM MM, but inferior to WM, which generally has an indolent course [28,29].

Therapeutically, patients respond to conventional MM regimens including proteasome inhibitors, immunomodulatory agents, and ASCT when feasible. Anti-CD20 therapy (e.g., rituximab) is ineffective due to the CD20^−^ immunophenotype of IgM MM, reinforcing the distinction from WM [28,29]. In a multicenter analysis from the CIBMTR, Reece et al. showed that patients with IgM MM had 3-year progression-free and overall survival rates of 47% and 68%, respectively—outcomes comparable to IgG and IgA myeloma, underscoring that ASCT is a viable option for IgM MM patients [35]. Notably, this supports that despite its rarity, IgM MM responds well to modern myeloma-directed therapies.

IgM MM is a rare but clinically significant subtype of MM that requires careful distinction from WM through integrated clinical, radiologic, immunophenotypic, and molecular assessment. Hallmark features such as t(11;14) translocation, absence of MYD88 L265P mutation, and presence of lytic bone lesions are central to diagnosis. Although outcomes with modern MM-directed therapies, including ASCT, appear comparable to other subtypes, important gaps remain. Future research should explore the molecular heterogeneity of IgM MM, particularly the biological implications of t(11;14) in this context, and whether targeted strategies such as BCL-2 inhibition (e.g., venetoclax) offer therapeutic advantages. Additionally, prospective studies and real-world data registries are needed to establish evidence-based guidelines for this understudied and diagnostically challenging plasma cell malignancy.

## 5. Non-Secretory Multiple Myeloma (NSMM)

Non-secretory multiple myeloma (NSMM) (Table 4) is a rare and diagnostically challenging subtype of multiple myeloma (MM), historically defined as lacking detectable monoclonal protein (M-protein) in both serum and urine using conventional electrophoresis. It accounts for approximately 1–5% of all MM cases and encompasses a heterogeneous group with distinct biological and clinical features [36,37]. With advancements in diagnostic technologies, particularly serum free light chain (FLC) assays and sensitive immunofixation techniques, the definition of NSMM has evolved, revealing a spectrum that includes truly non-secretory, oligosecretory, and hyposecretory forms [38,39].

NSMM can be subclassified into two main categories: [1] oligosecretory myeloma, where M-protein levels are detectable only by highly sensitive methods such as FLC assays or immunofixation, and [2] true NSMM, which lacks detectable immunoglobulin production even by the most sensitive assays [36,40,41]. The latter is exceedingly rare and may result from defective immunoglobulin synthesis or secretion at the cellular level [39]. The diagnostic approach requires a combination of bone marrow biopsy, imaging, and advanced immunological assays [40,41]. Some cases initially classified as non-secretory are reclassified as oligosecretory with sensitive testing—commonly referred to as “the missing M-band” [42]. Despite these advances, a major ongoing challenge in NSMM is the optimal assessment of treatment response, especially in the absence of measurable M-protein. The integration of minimal residual disease (MRD) assessment using next-generation flow cytometry or sequencing, along with advanced imaging modalities such as PET-CT and whole-body MRI, is reshaping response criteria in MM.

The clinical presentation of NSMM mirrors that of secretory MM, with patients often exhibiting anemia, bone lesions, renal dysfunction, or hypercalcemia. Marked hypogammaglobulinemia is usually seen in NSMM. However, due to the absence of measurable M-protein, diagnosis is often delayed, and disease monitoring becomes more reliant on imaging (MRI, PET-CT) and bone marrow evaluation [38,40,43]. Studies indicate that NSMM may present with a lower tumor burden at diagnosis, and some cohorts suggest comparable or even superior survival to secretory MM when treated with modern agents [41,42]. Conversely, outcomes may be worse for truly non-secretory cases where disease tracking is particularly difficult, though evidence is limited [44].

Therapeutic strategies for NSMM largely mirror those of conventional MM, including proteasome inhibitors, immunomodulatory drugs, monoclonal antibodies, and autologous stem cell transplantation [37,38,42]. Notably, patients benefit from novel-agent-based induction therapies similar to their secretory counterparts. However, monitoring treatment response poses a significant challenge. In the absence of measurable paraprotein, response assessments must rely on serial bone marrow examinations and imaging, which can limit sensitivity and delay detection of relapse [38,41].

Recent studies suggest that the cytogenetic risk profile of NSMM is comparable to that of secretory MM, with high-risk abnormalities such as del(17p), t(4;14), and gain(1q) occurring at similar frequencies [45]. These markers retain prognostic value and should guide risk-adapted therapy. Importantly, cytogenetic evaluation is essential in NSMM due to the lack of measurable serum biomarkers.

NSMM represents a biologically diverse spectrum of plasma cell disorders. The evolution of diagnostic methods, particularly FLC assays and functional imaging, has significantly improved detection and reclassification of cases previously labeled as “non-secretory”. Despite the inherent challenges in disease monitoring, patient outcomes have improved in the era of novel therapeutics. Key research priorities include the development of sensitive, non-invasive biomarkers for disease monitoring and relapse detection, as well as elucidation of the molecular mechanisms underlying true non-secretory behavior. Integrating dynamic imaging modalities with genomic profiling may further enhance response assessment and personalize treatment strategies in this rare and diagnostically elusive form of myeloma.

## 6. Plasma Cell Leukemia (PCL)

Plasma cell leukemia (PCL) (Table 5) is a rare and aggressive plasma cell disorder defined by the presence of circulating plasma cells in the peripheral blood. Traditionally, the diagnosis required either an absolute plasma cell count exceeding 2 × 10⁹/L or plasma cells comprising more than 20% of peripheral blood leukocytes. However, newer diagnostic criteria now recognize >5% circulating plasma cells as sufficient for diagnosis, reflecting improved detection methods and prognostic relevance. PCL can manifest either as primary PCL (de novo) or as secondary PCL (sPCL), which arises from the progression of previously diagnosed multiple myeloma (MM) [46,47,48,49].

PCL constitutes 1–4% of all plasma cell disorders and is more frequently observed in younger patients compared to MM [49,50]. Clinically, PCL presents with aggressive features including hepatosplenomegaly, lymphadenopathy, high tumor burden, anemia, thrombocytopenia, and renal impairment. Central nervous system and extramedullary involvement are more common than in MM [46,51].

Genomic studies have demonstrated that PCL harbors high-risk cytogenetic features such as del(17p), t(11;14), t(14;16), and complex karyotypes, which contribute to its aggressive behavior [52,53,54]. A genome-wide analysis highlighted significant transcriptional changes and chromosomal imbalances distinguishing PCL from MM [53]. The International Myeloma Working Group (IMWG) and subsequent studies emphasized that primary PCL (pPCL) has a distinct molecular signature and carries a worse prognosis [49,54].

In 2021, the International Myeloma Working Group (IMWG) lowered the PCL diagnostic threshold to >5% circulating plasma cells, reflecting similar aggressive behavior to the prior ≥20% cutoff [55]. pPCL tends to be more genetically unstable and aggressive compared to sPCL, with poorer outcomes if not promptly diagnosed and treated [47,48].

Treatment of PCL has evolved significantly with the advent of proteasome inhibitors, immunomodulatory agents, and monoclonal antibodies. Initial therapy typically includes bortezomib-based regimens combined with dexamethasone and an alkylating agent or immunomodulator [48,56]. The incorporation of bortezomib has led to improved response rates and prolonged survival, particularly in pPCL [56].

Autologous stem cell transplantation (ASCT) is commonly recommended for eligible patients with PCL after induction therapy; however, the durability of response remains limited, with many patients relapsing early [48,57]. To enhance disease control, consolidation strategies such as tandem ASCT or allogeneic hematopoietic cell transplantation (allo-HCT) are increasingly considered. A retrospective study analyzing 16 PCL patients undergoing either ASCT or allo-HCT reported that maintenance therapy was administered to 56% of patients post-ASCT [58]. The study found that patients receiving allo-HCT had a median progression-free survival (PFS) of 18 months compared to 6 months in the ASCT group, suggesting a potential benefit of allo-HCT in prolonging disease control [58]. Similarly, Lawless et al. demonstrated that patients undergoing ASCT followed by allo-HCT (auto-allo transplantation) had significantly improved PFS after 100 days compared to those receiving a single ASCT [59]. These findings support the role of tandem transplantation strategies, particularly for patients who fail to achieve complete remission prior to the first transplant.

Maintenance therapy post-transplant has also been explored as a means to sustain remission. In the aforementioned retrospective study, maintenance regimens such as lenalidomide, pomalidomide, and combinations with proteasome inhibitors were utilized [59]. However, no statistically significant association between maintenance therapy and improved PFS or overall survival (OS) was observed, likely due to the small sample size [59].

Novel agents, including anti-CD38 monoclonal antibodies (e.g., daratumumab), venetoclax for t(11;14) subsets, and CAR T-cell therapies, are being investigated for refractory PCL and offer hope for improved survival [60,61,62].

Despite therapeutic advances, the prognosis of PCL remains poor, with median overall survival of approximately 7–12 months in most series, though novel agents may improve outcomes [46,49,50]. Factors associated with poor outcomes include high LDH, elevated β2-microglobulin, extramedullary disease, and adverse cytogenetics [50,54,63]. A recent analysis showed that the presence of extraosseous plasmacytomas significantly worsened survival outcomes in pPCL patients [63].

PCL remains one of the most aggressive and therapeutically challenging plasma cell malignancies. While recent advances, including proteasome inhibitors, immunomodulatory agents, anti-CD38 monoclonal antibodies, and transplant strategies, have modestly improved outcomes, overall survival remains limited, particularly in primary PCL. Early identification using revised diagnostic thresholds and prompt initiation of intensive therapy are critical. Key future research directions include prospective evaluation of risk-adapted tandem or allo-HCT strategies, and exploration of the molecular vulnerabilities unique to PCL, such as transcriptional dysregulation and epigenetic alterations. Furthermore, systematic investigation of novel immunotherapies, such as CAR T cells, bispecific antibodies, and venetoclax in t(11;14)-positive disease, is urgently needed to define their role in frontline or salvage settings. International registries and multicenter clinical trials will be vital to generate evidence-based guidelines and improve outcomes in this ultra-high-risk population.

## 7. Heavy Chain Disease (HCD)

Heavy chain disease (HCD) (Table 6) is a rare and heterogeneous group of B-cell lymphoproliferative disorders characterized by the production of truncated, monoclonal immunoglobulin heavy chains without associated light chains. The condition is subclassified based on the isotype of the heavy chain involved: gamma (γ-HCD), alpha (α-HCD or Seligmann’s disease), and mu (μ-HCD) [64,65]. Among them, γ-HCD is the most commonly reported variant in Western countries [65,66].

HCD is characterized by the production of truncated immunoglobulin heavy chains without light chains due to defects in the variable region and constant domain assembly or somatic mutations during B-cell development [64,67]. These truncated chains escape normal quality control mechanisms within the endoplasmic reticulum and are secreted into serum without light chains [68].

γ-HCD exhibits marked clinical heterogeneity, presenting as either MGUS, a chronic lymphoproliferative disorder, or overt high-grade lymphoma [65,69]. The median age of diagnosis is around 65 years, with a slight female predominance [66].

Patients may present with lymphadenopathy, splenomegaly, constitutional symptoms (fever, weight loss, fatigue), and autoimmune phenomena, including associations with systemic lupus erythematosus (SLE) and rheumatoid arthritis [70]. HCD patients may also have renal complications, such as cast nephropathy [71].

Wahner-Roedler et al. reviewed 23 cases of γ-HCD, highlighting its variable course—ranging from indolent disease to aggressive lymphomas, particularly diffuse large B-cell lymphoma (DLBCL) [66]. Histologic analysis frequently reveals lymphoplasmacytic, marginal zone, or DLBCL-like morphology [69,72]. In some cases, no discernible tissue involvement is seen despite the presence of the monoclonal protein. Bieliauskas et al. found that among 13 γ-HCD cases, over half were associated with defined lymphomas, and a few with autoimmune disease without overt neoplasia [69].

Immunohistochemistry typically shows cytoplasmic expression of heavy chains without associated light chains, often with positive staining for CD19, CD20, and CD79a [69].

Autoimmune disorders, particularly SLE, are strongly associated with γ-HCD, possibly due to chronic antigenic stimulation and B-cell dysregulation [65,67]. Fermand et al. presented a unique case of γ-HCD initially mimicking autoimmune pathology that later progressed to DLBCL [65]. Although rare, renal involvement has been described. A recent case reported cast nephropathy due to γ-HCD, mimicking light chain cast nephropathy, emphasizing the diagnostic challenges in atypical presentations [64].

Diagnosis involves serum and urine protein electrophoresis with immunofixation, quantification of immunoglobulin isotypes, bone marrow biopsy, and flow cytometry. Tissue biopsy where lymphoma is suspected should be considered. Mass spectrometry offers a highly sensitive method for identifying heavy chain fragments in atypical cases [64].

There is no standard treatment for γ-HCD. Management depends on the associated lymphoproliferative disorder. Indolent cases may be monitored without immediate therapy [66,69]. DLBCL-type γ-HCD is treated using R-CHOP or other anthracycline-based regimens [73]. In the presence of autoimmune disease, immunosuppressive agents or corticosteroids may be used initially [65]. The prognosis varies considerably. Patients with isolated γ-HCD and no underlying lymphoma may remain stable for years, while those with aggressive lymphomas have poorer outcomes [64,66,69].

γ-HCD represents a rare and clinically heterogeneous B-cell neoplasm that spans a spectrum from indolent gammopathies to aggressive lymphomas. Its diagnostic complexity is heightened by its potential to mimic autoimmune conditions and by the absence of light chains on standard assays. A high index of suspicion and use of advanced tools such as immunofixation, tissue immunophenotyping, and mass spectrometry are crucial for accurate identification. Future research should aim to elucidate the molecular pathogenesis of heavy-chain-only secretion, including the role of somatic mutations, and chronic immune stimulation. In addition, prospective studies are needed to define risk stratification criteria; therapeutic decision-making frameworks; and the potential efficacy of novel agents, such as B-cell targeted therapies or anti-CD20 monoclonals, in various clinical presentations of γ-HCD. Given the rarity of this entity, international registry efforts and collaborative case series will be instrumental in guiding evidence-based management and improving long-term outcomes.

## 8. Conclusions

Rare plasma cell disorders, including IgD, IgE, and IgM MM, NSMM, PCL, and HCD, pose substantial diagnostic challenges due to their biological heterogeneity, atypical clinical features, and frequent lack of detectable monoclonal proteins on conventional assays. The rarity of these entities often leads to delayed or missed diagnoses, particularly in cases like NSMM and HCD where standard serum electrophoresis and immunofixation may yield false-negative results. Accurate classification increasingly depends on the integration of advanced diagnostic tools, including serum free light chain assays, mass spectrometry, cytoplasmic immunoglobulin staining, immunophenotyping, and next-generation sequencing. However, these tools are not uniformly available or standardized across clinical settings, creating variability in diagnostic precision.

Future efforts must prioritize the development of diagnostic algorithms tailored to rare subtypes, validation of surrogate disease markers for response assessment in non-secretory and atypical presentations, and the use of functional imaging and MRD techniques to guide treatment decisions. Establishing international registries and embedding these patients in subtype-specific or biomarker-driven clinical trials will be essential to deepen our understanding of disease biology and therapeutic vulnerabilities. Ultimately, a unified approach combining standardized diagnostics, molecular stratification, and collaborative clinical research is critical to improving outcomes for these diagnostically elusive and underrepresented plasma cell malignancies.

## Figures and Tables

**Table 1:** Clinical and biological overview of IgD multiple myeloma.

Category	Key Findings
Epidemiology	Accounts for 1–2% of MM; male predominance; younger age (50–60 years).
Clinical presentation	Commonly presents with renal impairment, bone lesions, amyloidosis, hypercalcemia, and Bence Jones proteinuria.
Diagnosis challenges	Delayed diagnosis due to low serum IgD and absent M-protein spike; immunofixation and free light chain assays crucial.
Light chain association	Predominantly lambda light chain restriction (>85%); kappa association is rare
Cytogenetic abnormalities	Frequent del(13q), 1q21 gain, IGH rearrangements; t(11;14) common; t(14;16) and del(17p) variably reported.
Prognosis (historical)	Median OS 13–21 months due to late diagnosis and renal dysfunction.
Prognosis (modern)	Median OS improved to 48–50+ months with novel agents and ASCT.
Prognostic factors	Renal dysfunction and high β2-microglobulin levels predict worse OS.
Therapeutic advances	Novel agents (bortezomib, lenalidomide, thalidomide) and ASCT improved responses (up to 89% response rate).
Unmet needs	Late diagnosis, variable cytogenetics, high-risk features continue to impact survival; need for risk stratification and trials.

**Table 2:** Clinical and biological overview of IgE multiple myeloma.

Category	Key Findings
Epidemiology	Rarest MM subtype (<0.1%); described in 1967; predominantly male; younger onset.
Clinical behavior	Aggressive course; frequent extramedullary disease, plasma cell leukemia, and poor survival.
Clinical presentation	Features similar to MM (anemia, bone pain, hypercalcemia); more frequent hyperviscosity and PCL.
Diagnosis challenges	Low serum IgE causes false negatives in electrophoresis; immunofixation and sFLC assays critical.
Light chain association	Strong lambda light chain bias; consistent with transcriptional data from IgE-secreting plasma cells.
Rare presentations	Reported dual IgE/IgA monoclonal proteins with shared clonal origin.
Evolution and progression	May evolve from MGUS; some cases progress to secondary PCL, median survival 1–2 months.
Complications	Hyperviscosity syndrome and elevated CA125 reported in absence of solid tumors.
Molecular features	IgE plasma cells are transcriptionally unique; high ER stress, TACI, and BCMA expression.
Genomic insights	High mutation burden and tumor-reactive T cells suggest potential for personalized immunotherapy.
Treatment and prognosis	No IgE-specific protocols; treated as conventional MM; survival remains shorter than IgG/IgA MM.
Research and future directions	Case reports and immunogenetic studies offer insights; future strategies may improve outcomes.

**Table 3:** Clinical and biological overview of IgM multiple myeloma.

Category	Key Findings
Epidemiology	Extremely rare (<0.5% of MM); overlaps clinically with Waldenström macroglobulinemia (WM).
Clinical features	Presents with CRAB features; IgM monoclonal protein present; may include WM-like symptoms.
Diagnostic criteria	≥10% plasma cells, IgM M-protein, CRAB criteria, bone lesions, and absence of MYD88 mutation.
Light chain association	Typically kappa-restricted; lambda chains are less common and should prompt diagnostic re-evaluation.
Cytogenetics	Commonly harbors t(11;14)(q13;q32); cyclin D1 dysregulation seen in ~39–50% of cases.
Immunophenotype	CD138+, cyclin D1+; lacks CD20, CD19, CD56, CD117—distinguishes from WM clones.
Differentiation from WM	MYD88 L265P mutation absent; histology reveals plasma cells (vs. lymphoplasmacytic in WM).
Survival and prognosis	Median OS ~ 61 months; comparable to IgG/IgA MM, inferior to indolent WM outcomes.
Therapeutic strategies	Responds to MM regimens (PI, IMiDs, ASCT); anti-CD20 (e.g., rituximab) not effective.
Case reports and variants	Reports of rare presentations (e.g., spinal cord compression); diagnostic reclassification not uncommon.
Clinical implications	Accurate distinction from WM is critical for therapy; integrated diagnostic approach essential.

**Table 4:** Clinical and biological overview of non-secretory multiple myeloma.

Category	Key Findings
Epidemiology	Accounts for 1–5% of MM; initially defined by absence of M-protein in serum/urine.
Definition and evolution	Definition has evolved with FLC assays; now includes truly non-secretory, oligosecretory, and hyposecretory forms.
Subtypes	Two main types: oligosecretory (detectable by FLC/immunofixation) and true non-secretory (no detectable Ig).
Diagnostic challenges	Diagnosis requires bone marrow, imaging, and advanced assays; ’missing M-band’ cases reclassified with repeat testing.
Clinical features	Presents with CRAB features; diagnosis often delayed due to lack of measurable M-protein.
Prognosis	May have lower tumor burden at diagnosis; survival comparable or better than secretory MM in some studies.
Therapeutic approaches	Treatment mirrors MM: PI, IMiDs, antibodies, ASCT; modern regimens benefit NSMM patients.
Response monitoring	Response assessment challenging; relies on bone marrow and imaging rather than serum biomarkers.
Cytogenetics	Cytogenetic risks (del(17p), t(4;14), gain(1q)) occur at similar rates as in secretory MM.
Future directions	Research needed on molecular markers and surrogate endpoints to guide therapy and monitoring.

**Table 5:** Clinical and biological overview of plasma cell leukemia (PCL).

Category	Key Findings
Definition	Defined by >5% circulating plasma cells in peripheral blood (IMWG 2021 criteria).
Epidemiology	Accounts for 1–4% of plasma cell malignancies; often affects younger patients.
Clinical features	Aggressive presentation with hepatosplenomegaly, CNS/extramedullary disease, anemia, and renal dysfunction.
Genomic and cytogenetic features	High-risk cytogenetics (e.g., del(17p), t(11;14), t(14;16), complex karyotypes); distinct transcriptional profile.
Revised diagnostic criteria	IMWG revised diagnostic cutoff from 20% to >5% circulating plasma cells based on recent evidence.
Treatment strategies	Initial therapy typically includes bortezomib + dexamethasone + alkylator/IMiD; improved response with novel agents.
Transplantation approaches	ASCT used in eligible patients; allo-HCT and tandem transplant (auto-allo) associated with better PFS in some studies.
Maintenance therapy	Maintenance therapy (lenalidomide, pomalidomide, PIs) explored post-transplant; data remain inconclusive.
Emerging therapies	Anti-CD38 mAbs, venetoclax (t(11;14)), CAR T-cells under investigation for refractory disease.
Prognosis	Prognosis poor (median OS 7–12 months); adverse factors include high LDH, β2-M, extramedullary disease.
Future directions	Further research on early diagnosis, novel agents, and consensus criteria urgently needed.

**Table 6:** Clinical and biological overview of gamma heavy chain disease (γ-HCD).

Category	Key Findings
Definition	Rare B-cell disorder with production of truncated heavy chains lacking light chains.
Subtypes	Includes γ-HCD (most common), α-HCD (Seligmann’s disease), and μ-HCD.
Pathogenesis	Defective assembly of immunoglobulin chains leads to secretion of free heavy chains.
Epidemiology	Median age ~65 years, slight female predominance; γ-HCD most common in Western populations.
Clinical presentation	Variable symptoms: lymphadenopathy, splenomegaly, fever, weight loss, autoimmune disease.
Histopathology	Histology shows lymphoplasmacytic, marginal zone, or DLBCL-like features.
Immunophenotype	Cytoplasmic heavy chain expression; CD19+, CD20+, CD79a+; lacks light chains.
Autoimmune associations	Frequently associated with SLE and RA; some cases initially mimic autoimmune disease.
Renal involvement	Rare cases show renal cast nephropathy similar to light chain disease.
Diagnostic approach	Requires serum/urine electrophoresis, immunofixation, mass spectrometry, and biopsy.
Treatment strategies	No standard therapy; managed per associated lymphoma or autoimmune condition.
Prognosis	Highly variable; indolent forms may be stable; aggressive forms have poor prognosis.
Clinical implications	Often misdiagnosed; awareness and advanced diagnostics critical for accurate classification.

## Data Availability

This article is a review of previously published literature. No new data were generated or analyzed in the preparation of this manuscript. All data supporting the conclusions are available in the cited primary sources.
